# Differential Contribution of PB1-F2 to the Virulence of Highly Pathogenic H5N1 Influenza A Virus in Mammalian and Avian Species

**DOI:** 10.1371/journal.ppat.1002186

**Published:** 2011-08-11

**Authors:** Mirco Schmolke, Balaji Manicassamy, Lindomar Pena, Troy Sutton, Rong Hai, Zsuzsanna T. Varga, Benjamin G. Hale, John Steel, Daniel R. Pérez, Adolfo García-Sastre

**Affiliations:** 1 Department of Microbiology, Mount Sinai School of Medicine, New York, New York, United States of America; 2 Department of Veterinary Medicine, University of Maryland, College Park, Maryland, United States of America; 3 Department of Microbiology and Immunology, School of Medicine, Emory University, Rollins Research Center, Atlanta, Georgia, United States of America; 4 Institute of Global Health and Emerging Pathogens, Mount Sinai School of Medicine, New York, New York, United States of America; 5 Department of Medicine, Mount Sinai School of Medicine, New York, New York, United States of America; Johns Hopkins University - Bloomberg School of Public Health, United States of America

## Abstract

Highly pathogenic avian influenza A viruses (HPAIV) of the H5N1 subtype occasionally transmit from birds to humans and can cause severe systemic infections in both hosts. PB1-F2 is an alternative translation product of the viral PB1 segment that was initially characterized as a pro-apoptotic mitochondrial viral pathogenicity factor. A full-length PB1-F2 has been present in all human influenza pandemic virus isolates of the 20^th^ century, but appears to be lost evolutionarily over time as the new virus establishes itself and circulates in the human host. In contrast, the open reading frame (ORF) for PB1-F2 is exceptionally well-conserved in avian influenza virus isolates. Here we perform a comparative study to show for the first time that PB1-F2 is a pathogenicity determinant for HPAIV (A/Viet Nam/1203/2004, VN1203 (H5N1)) in both mammals and birds. In a mammalian host, the rare N66S polymorphism in PB1-F2 that was previously described to be associated with high lethality of the 1918 influenza A virus showed increased replication and virulence of a recombinant VN1203 H5N1 virus, while deletion of the entire PB1-F2 ORF had negligible effects. Interestingly, the N66S substituted virus efficiently invades the CNS and replicates in the brain of Mx+/+ mice. In ducks deletion of PB1-F2 clearly resulted in delayed onset of clinical symptoms and systemic spreading of virus, while variations at position 66 played only a minor role in pathogenesis. These data implicate PB1-F2 as an important pathogenicity factor in ducks independent of sequence variations at position 66. Our data could explain why PB1-F2 is conserved in avian influenza virus isolates and only impacts pathogenicity in mammals when containing certain amino acid motifs such as the rare N66S polymorphism.

## Author Summary

Influenza A viruses can infect avian and mammalian hosts. Human infections with seasonal influenza virus strains are usually confined to the respiratory tract and are cleared within days by the immune system. In contrast, highly pathogenic avian influenza viruses can spread throughout the whole body, usually resulting in multi-organ failure and even death in immune competent hosts. Here, we investigated the species-specific function of an influenza A virus protein, PB1-F2, that is highly conserved in avian influenza virus strains but which is lost in many isolates from mammalian hosts. Our data indicate that PB1-F2 allows successful spreading of the virus throughout the body in experimentally infected ducks. In contrast, PB1-F2 does not contribute to the severity of HPAIV infections in mice. Nevertheless, a polymorphism at position 66 of PB1-F2 (N66S) that was found in the devastating 1918 pandemic virus and in several early H5N1 HPAIV isolates clearly increased pathogenicity of a HPAIV influenza virus in mice. Our findings might explain why the whole PB1-F2 ORF is conserved in avian influenza viruses, since it contributes to viral dissemination and pathogenicity, but can be lost in mammalian hosts as it has minimal effects on virulence.

## Introduction

Direct bird-to-human transmission of highly pathogenic avian influenza A viruses (HPAIV) of the H5N1 subtype was first reported in 1997 [1]. Since then, 518 human cases have been confirmed (WHO: updated January 20^th^, 2011). Unlike seasonal influenza A viruses, HPAIVs do not yet have the ability to spread directly from human-to-human, a likely consequence of multiple species barriers, including their preferential attachment to alpha 2,3-linked sialic acids which are rarely present in the upper regions of the human respiratory tract [2]. However, human infections with these viruses often clinically manifest with an unusual hyperactivation of the host immune response, vast overproduction of cytokines and chemokines, severe inflammatory response syndrome, fever, pneumonia, pulmonary hemorrhage, acute respiratory distress syndrome, lymphopenia, prominent hemophagocytosis, disseminated intravascular thrombosis, diarrhea and multiorgan failure [3], [4], [5], [6], [7]. Despite the low number of cases so far, the overall human mortality rate of HPAIV (∼60%, WHO: updated January 20^th^, 2011) is of serious concern, and the possibility that these HPAIVs might reassort with a circulating human strain, thereby potentially integrating lethality and transmissibility, is a continuous public-health risk.

Waterfowl, particularly ducks, are an important natural reservoir for a variety of influenza A viruses [8]. Infections with low pathogenic avian influenza A virus are usually asymptomatic in ducks [9]. The virus mainly replicates in enterocytes in the digestive tract of infected animals and is shed in the feces [10], [11]. Even most HPAIV infections only cause mild clinical signs in ducks and immune competent animals usually recover from infection [12]. In contrast, chickens often die abruptly by infection with HPAIV. Despite similar multi-organ tropism in ducks and chicken, HPAIV apparently replicates better in chicken, as higher viral titers are commonly observed [12]. Exceptions to this are clade 2.2 H5N1 HPAIVs, which cause death in experimentally infected ducks [13].

PB1-F2 was identified ∼10 years ago during the screening of MHC presented epitopes of alternative ORF gene products from influenza A virus infected cells [14]. This 90 amino acid polypeptide is translated from the PB1 mRNA from an alternative start codon in the +1 reading frame, approximately 100 base pairs downstream of the PB1 start codon. Initially PB1-F2 was characterized as a pro-apoptotic peptide, integrating into the inner mitochondrial membrane and disrupting mitochondrial membrane potential, apparently with a preference for monocytic cells [14], [15], [16], [17]. In this regard, later studies showed that PB1-F2 can interact with the mitochondrial membrane proteins VDAC-1 and ANT3 [18], both of which are involved in maintaining the mitochondrial membrane potential [19]. For the mouse-adapted human H1N1 isolate A/PR/8/1934 (PR8), PB1-F2 can increase the viral polymerase function, presumably due to its interaction with the polymerase subunit PB1 [20]. Recent studies have confirmed both the pro-apoptotic and polymerase enhancing functions of PB1-F2, but have clearly shown that these activities are highly dependent upon the specific virus isolate used [21].

With parallels to the current sporadic bird-to-human transmissions of H5N1 HPAIV, the causative agent of the devastating 1918 “Spanish flu” (1918 H1N1 virus) was proposed to have originated in humans as a direct transmission from birds. The PB1-F2 of this virus is unusual in that it contains a serine at position 66 instead of asparagine. A recombinant mutant 1918 virus with a substitution of asparagine for serine at position 66 (S66N) showed increased MLD_50_, which was approximately two to three orders of magnitude higher than for the wild type recombinant virus [22]. The molecular mechanism for enhanced pathogenesis caused by the 66S polymorphism is not fully understood. However, recent experiments with PB1-F2 derived peptides suggest that the C-terminus of PB1-F2, which includes residue 66, can induce proinflammatory cytokines [23]. In addition, PB1-F2 of 1918:H1N1 has been shown to increase secondary bacterial infections of mice [24].

Descendents of the 1918 H1N1 virus still circulate in humans to date, but soon after their introduction into humans they acquired an asparagine at position 66, and in the early 1950s much of the PB1-F2 ORF was lost by introduction of a premature stop codon at position 58, thus leaving the polypeptide without its C-terminal mitochondrial targeting sequence. Furthermore, an identical truncation occurred independently in human isolates after the reintroduction of H1N1 viruses expressing a 90aa PB1-F2 in the late 1970s. For human H2N2 and H3N2 viruses, the original pandemic viruses had a PB1 gene derived by reassortment from an avian influenza virus strain, containing a full-length PB1-F2 ORF. Recently published data suggest that at least the PB1-F2s encoded by current human H3N2 viruses might not be functional [21]. Thus, it has been suggested that in humans there is no selective pressure to maintain full-length and/or functional PB1-F2 and that PB1-F2 might have a different role in mammalian versus avian hosts [25]. In this regard, reintroduction of the PB1-F2 ORF by reverse genetics into the novel 2009 pandemic H1N1 virus did not significantly increase pathogenicity of this virus in mouse [26]. In stark contrast to the situation in human influenza A viruses, full length PB1-F2 ORFs are highly conserved in viruses isolated from avian species [25], especially waterfowl. For example, in duck virus isolates the prevalence of full length PB1-F2 is almost 96%. Nevertheless, the role of PB1-F2 in avian species is unknown and comprehensive studies in avian systems have been limited.

The PB1-F2s of all human influenza A virus pandemics of the last century were derived from avian strains. In this study, we compared effects of PB1-F2 expression and sequence variation at position 66 on viral pathogenesis and host response in two prototype mammalian and avian model organisms, mice and ducks. We sought to delineate host dependent functions of PB1-F2 from a highly pathogenic H5N1 avian influenza virus that has crossed the bird-mammal species barrier and caused severe infections in both host systems.

## Materials and Methods

### Ethics Statement

This study was carried out in strict accordance with recommendations in the Guide for the Care and Use of Laboratory Animals of the National Institutes of Health. All mouse procedures were approved by Institutional Animal Care and Use Committee (IACUC) of Mount Sinai School of Medicine and performed in accordance with the IACUC guidelines (Protocol # LA08-00594: “Contribution of NS1 to pathogenicity and evasion of innate immunity”). Duck experiments were conducted under ABSL-3 conditions in a USDA approved facility and performed according to protocol R-09-93 “Transmissibility of Influenza A Viruses”, approved by the Institutional Animal Care and Use Committee of the University of Maryland.

### Antibodies and Reagents

A customized polyclonal serum against the N-terminal 37aa of A/Viet Nam/1203/2004 PB1-F2 was raised in rabbits (Genescript). Monoclonal mouse anti-influenza A virus anti-NP (clone 28D8) was generated against RNPs of A/Puerto Rico/8/1934 in the hybridoma facility of MSSM, NY. Mouse monoclonal antibody against prohibitin was purchased from Abcam (clone II-14-10).

### Viruses and Cells

All recombinant influenza A/Viet Nam/1203/2004 (VN1203) viruses and the low pathogenic version thereof were described previously [27]. The PB1-F2 deletion mutant of this virus was generated by substitution of bases T96C (in PB1-F2-ORF: ATG->ACG), C129G (TCA->TGA), C198G (TCA->TGA), T210C (ATG->ACG) and T231C (ATG->ACG) in the PB1 ORF of pPol1-PB1 A/Viet Nam/1203/2004, thus eliminating all 3 ATGs and introducing 2 additionally two stop codons in the PB1-F2 ORF, using the Quick Change Site directed mutagenesis kit (Stratagene) according to the manufacturer's instructions. The PB1-F2 N66S mutant was generated by introducing the A291G substitution (in PB1-F2-ORF: AAT->AGT). None of the mutations result in non-synonymous changes in the ORF of PB1 or N40. The same mutations were introduced into a low pathogenic mutant of VN1203 lacking the multi-basic cleavage site of HA (HAlo). All viruses were plaque purified and successful PB1-F2 mutagenesis was confirmed by sequencing the viral RNA of stocks used for infection experiments. No additional mutations were found in any other gene segment of the recombinant viruses .

Madin Darby canine kidney (MDCK) cells, murine lung epithelial adenoma cells (LA-4) and duck embryonic fibroblasts (DEF) were purchased from ATCC and cultured according to the manufacturer's instructions. Murine bone marrow derived macrophages (BMDMs) were generated and cultured as described previously [28]. Murine bone marrow derived dendritic cells (BMDDCs) were isolated as BMDMs, but differentiated in the presence of recombinant murine GM-CSF at 20 ng/ml (R&D) [29].

### Animal Models

C57/BL/6 mice were purchased from Jackson Laboratories. C57/BL/6/A2G-Mx1, a mouse line expressing functional Mx1, was kindly provided by Peter Staeheli, University of Freiburg, Germany. These mice were generated in a fashion similar to the previously described BALBc/A2G-Mx1 mice [30]. All viral infections of mice were performed in accordance with CDC and USDA guidelines in the ABSL-3+ facility of Mount Sinai School of Medicine, New York, NY. Briefly, mice were anesthetized by intraperitoneal injection of ketamine-xylazine and infected intranasally with 50 µl of virus diluted in PBS. The mice were monitored for clinical signs and weight loss daily. Animals were humanely euthanized upon reaching 75% of initial body weight. MLD_50_ was calculated according to the method of Reed and Muench. Viral lung titers were determined by standard plaque assay on MDCK cells [31].

Two-week old White Peking ducks (McMurray Hatchery, IA, USA) were randomly divided into four treatment groups (n = 16) and housed in separate isolators. Following the acclimatization period, animals were mock inoculated with PBS or infected through natural routes with 10^4^ pfu of the recombinant viruses diluted in 1 ml PBS (0.5 ml intratracheal, 0.3 ml into the nares, and 0.2 ml into the eyes). Clinical signs (depression, cyanosis of the skin, respiratory involvement, diarrhea, edema of the face and/or head, and neurological signs) were monitored and scored daily. A pathogenicity index was calculated as previously described [32]. Tracheal and cloacal swabs were taken on days 1, 3 and 5 post-infection. Viral load of multiple organs were determined on day 1 and 3 post infection using the TCID_50_ method as described previously [33].

### Quantitative PCR (qPCR) and Primer Sequences

Total RNA was isolated by Qiagen RNeasy Mini Kits (Qiagen) or TRIzol (Invitrogen) according to the manufacturer's instructions. 1–5 µg of total RNA was used for reverse transcription by oligo-dT priming in a 20 µl volume (1^st^ Strand cDNA synthesis kit, Roche). 0.5 µl of 1∶10 diluted cDNA, 2 µl of 10 µM gene specific primer mix (sequences for murine cDNAs, duck cDNAs and influenza segment 7 see Table 1) and 5 µl of 2x SYBR-Green qPCR Mastermix (Roche) were used for qPCR in a Light-Cycler480 (Roche). Mean N-fold expression levels of cDNA from three individual biological samples, each measured in triplicates, were normalized to 18S rRNA levels and calibrated to mock treated samples according to the 2^−ΔΔCT^ method [34].

**Table 1:** qPCR primers for murine and duck cDNAs and influenza segment 7.

*mouse gene*	*forward (5′ -3′)*	*reverse (5′ -3′)*
**IL1beta**	GCAACTGTTCCTGAACTCAACT	ATCTTTTGGGGTCCGTCAACT
**IL18**	GACTCTTGCGTCAACTTCAAGG	CAGGCTGTCTTTTGTCAACGA
**IFNbeta**	CAGCTCCAAGAAAGGACGAAC	GGCAGTGTAACTCTTCTGCAT
**Mx1**	GACCATAGGGGTCTTGACCAA	AGACTTGCTCTTTCTGAAAAGCC
**IFNgamma**	TGCTGATGGGAGGAGATGTCTAC	TTTCTTTCAGGGACAGCCTGTTAC
**IL6**	TGAGATCTACTCGGCAAACCTAGTG	CTTCGTAGAGAACAACATAAGTCAGATACC
**ISG15**	GGTGTCCGTGACTAACTCCAT	TGGAAAGGGTAAGACCGTCCT
**MIP1alpha**	CGAGTACCAGTCCCTTTTCTGTTC	AAGACTTGGTTGCAGAGTGTCATG
**MIP1beta**	AAGCTGCCGGGAGGTGTAAG	TGTCTGCCCTCTCTCTCCTCTTG
**IFNalpha1**	TCAAAGGACTCATCTGCTGCTTG	CCACCTGCTGCATCAGACAAC
**CCL5**	TTGACCCGTAAATCTGAAGCTAAT	TCACAGTCCGAGTCACACTAGTTCAC
**OAS1**	ATGGAGCACGGACTCAGGA	TCACACACGACATTGACGGC
**IL10**	GGGTTGCCAAGCCTTATCG	TCTCACCCAGGGAATTCAAATG
**CX3CL1**	GGACAACACCATTACTGTGCCTTAG	CACACAGGGATGGCTGAGTTCTAC
**IP10**	CCAAGTGCTGCCGTCATTTTC	GGCTCGCAGGGATGATTTCAA
**18S**	GTAACCCGTTGAACCCCATT	CCATCCAATCGGTAGTAGCG

*IAV segment*	*forward (5′ -3′)*	*reverse (5′ -3′)*
**influenza M**	AGATGAGTCTTCTAACCGAGGTCG	TGCAAAAACATCTTCAAGTCTCTG

*Duck gene*	*forward (5′ -3′)*	*reverse (5′ -3′)*
GAPDH	ATGTTCGTGATGGGTGTGAA	CTGTCTTCGTGTGTGGCTGT
IL6	GTGCGAGAACAGCATGGAGATG	GAATCTGGGATGACCACTTCATC
IFNB	CCTCAACCAGATCCAGCATT	GGATGAGGCTGTGAGAGGAG

### Immunofluorescence Microscopy

Immunofluorescence microscopy was performed using a Zeiss Axioplan fluorescence microscope and Axiovision image acquisition and analysis software at the MSSM-Microscopy Shared Resource Facility, supported with funding from NIH-NCI shared resources grant (5R24 CA095823-04), NSF Major Research Instrumentation grant (DBI-9724504) and NIH shared instrumentation grant (1 S10 RR0 9145-01).

### SDS-PAGE and Western Blot Analysis

SDS PAGE and western blot were performed as described previously [35]. Briefly, total cell lysates were obtained by collecting adherant and floating cells in disruption buffer containing 6 M urea, 2 M b-mercaptoethanol and 4% SDS. Genomic DNA was fragmented by brief sonication on ice. Lysates were not centrifuged before gel electrophoresis since this method of disruption does not yield a visible pellet after centrifugation. Total protein lysates were separated on 4–15% or 4–20% gradient Ready-Gels (Biorad) and transferred to PVDF membranes for western blot analysis.

### Polymerase Assay

The murine RNA-polymerase I driven negative sense luciferase reporter plasmid (pcDNA3.1-IVAR-Luc) was cloned by replacing the CMV promotor of pcDNA3.1 (Invitrogen) with bp -2150 - +1 of the murine 45S ribosomal RNA promotor. We used pMrTsp-9-T10 plasmid, kindly provided by Dr. Gernoth Längst, University of Regensburg, Germany, as template for amplification of murine RNA polymerase I promotor and terminator sequences [36]. A negatively orientated firefly luciferase coding sequence, flanked by influenza NP promotor elements was inserted downstream. The murine RNA Pol1 terminator of 45S ribosomal RNA was introduced downstream of the negatively oriented reporter gene. Murine 3T3 fibroblasts were transfected in 24 well format with 50–400 ng of pCAGGS PB1 (VN1203 WT or mutants PB1-F2 generated as described for the recombinant viruses), 100 ng pCAGGS-PB2 and -PA and 200 ng of pCAGGS-NP (all VN1203), 100 ng of pcDNA3.1-IVAR-Luciferase and 50 ng of pCMV-Renilla-luciferase, using Lipofectamin 2000 (Invitrogen) according to the manufacturers instructions. Cells were lysed 24 h post transfection according to the manufacturers instruction (Dual Luciferase Kit Promega). Firefly-Luciferase activity was normalized by Renilla activity. Samples were measured in independent biological triplicates.

### Statistical Analysis

All statistical analyses were performed using GraphPad Prism Software Version 5.00 (GraphPad Software Inc., San Diego, CA). Comparison between two treatment means was achieved using a two-tailed Student t-test, whereas multiple comparisons were carried out by analysis of variance (ANOVA) followed by Dunnett's test using the WT virus as the control. Survival analyses were carried out following the Kaplan Meier procedure. The differences were considered statistically significant at p<0.05.

## Results

PB1-F2 has been associated with a spectrum of functions within influenza A virus infected cells. Some of these functions are believed to be host cell type and/or virus strain specific. To comparatively study the role of a HPAIV PB1-F2 in both mammalian and avian hosts, we used reverse genetics to generate the wild-type recombinant VN/1203/04 (H5N1) (VN1203) virus (WT), together with a PB1-F2 deficient mutant (dF2) and a substitution mutant bearing a serine at position 66 (N66S). VN1203 was selected as this viral strain can efficiently infect both mice and ducks [37], [38], and causes severe disease in these species.

### PB1-F2 with the N66S Mutation Increases H5N1 Viral Replication in Murine Cells

**Figure 1:**
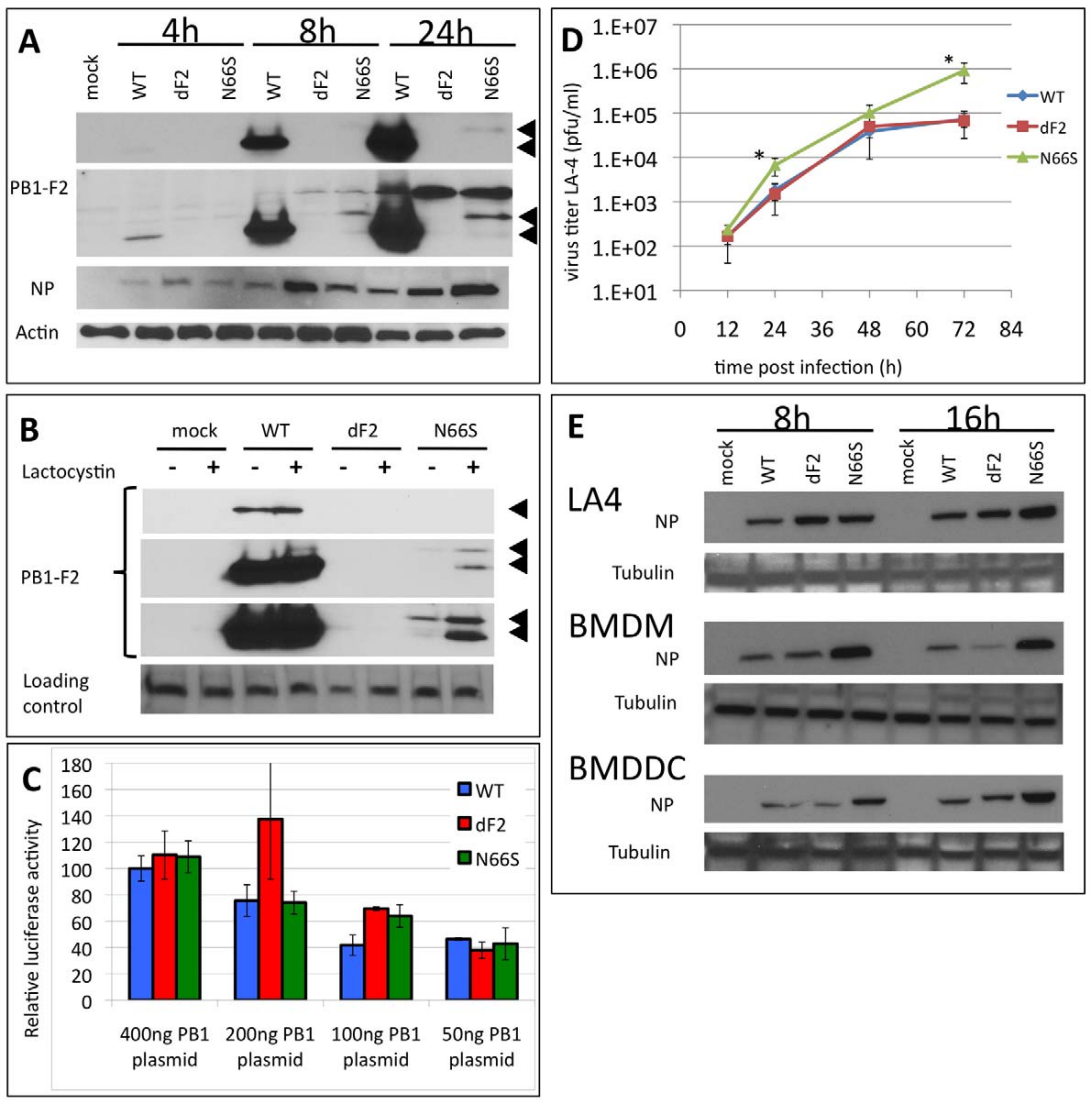
Effects of PB1-F2 expression on replication of A/Viet Nam/1203/2004 in murine cells in vitro. A) Time course of viral protein expression. Murine lung epithelial cells (LA-4) were infected with 2MOI of A/Viet Nam/1203/2004 wild type, dF2 or N66S. Cells were lysed after indicated time points. Whole cell extracts were analyzed by western blot with specific antibodies against PB1-F2 (upper two panels, short and long exposure, respectively, PB1-F2 is indicated by black arrows), viral nucleoprotein (NP) or beta-Actin. B) PB1-F2 expression in presence or absence of 10 µM lactocystin. LA4 were infected with 2MOI of A/Viet Nam/1203/2004 wild type, dF2 or N66S. Cells were lysed after 24 h. Whole cell extracts were analyzed by western blot with specific antibodies against PB1-F2 (upper three panels, short, medium and long exposure, respectively, PB1-F2 is indicated by black arrows), equal loading is indicated by an unspecific background band (lower panel). C) Polymerase Assay. Murine fibroblasts (3T3) were transfected with indicated amounts of pCAGGS-PB1 A/Viet Nam/1203/2004 wild type (blue), dF2 (red) or N66S (green) and constant amounts of PB2, PA, NP and reporter gene expressing plasmids as described in Material and Methods. Mean firefly-luciferase activity of three independent transfections normalized to renilla-luciferase activity is depicted. Error bars represent SD. No significant differences were detected. D) Multi-cycle growth curve of A/Viet Nam/1203/2004 wild type (blue), dF2 (red) or N66S (green) on LA-4. Cells were infected with 0.05MOI of virus. Viral supernatants were taken at the indicated time points. Viral titers were determined by standard plaque assay on MDCK in absence of trypsin. Mean pfu/ml of independent triplicates ±SD indicated by the error bar are depicted. *P*-values are indicated (*p<0.05) using WT as standard. E) Time course of viral protein expression in LA-4 (upper panel), bone marrow derived macrophages (BMDM, middle panel) and bone marrow derived dendritic cells (BMDDC). Cells were infected with 2MOI of A/Viet Nam/1203/2004 wild type, dF2 or N66S and lysed after indicated time points. Whole cell extracts were analyzed by western blot with specific antibodies against viral nucleoprotein (NP) and tubulin.

We first studied the replication kinetics of VN1203 WT, dF2 and N66S viruses in the murine lung epithelial adenoma cell-line LA-4. Although similar or higher levels of viral nucleoprotein (NP) were observed for the mutant viruses as compared to WT, the expression of the N66S mutant form of PB1-F2 was lower than that of WT and barely detectable at 24 h post infection (Fig. 1A, panel 2, long exposure). Treatment with 10 µM lactocystin resulted in increased levels of PB1-F2 N66S but not WT, suggesting specific proteasomal degradation of the mutant PB1-F2 (Fig. 1B). Interestingly, the N66S substituted PB1-F2 migrates slower in the gel, possibly indicating a posttranslational modification. Interestingly, the PB1-F2 deficient virus generates higher amounts of NP 4 h post infection. These levels adjust to WT at later timepoints, implying an early deregulation of protein expression. However, deletion of the PB1-F2 ORF does not change polymerase activity (Fig. 1C) or viral multi-cycle growth in these cells: the WT and PB1-F2 deficient viruses grow to identical titers. Notably, the N66S substituted virus grows to 10-fold higher titers than WT or dF2 after 72 h (Fig. 1D).

Previous studies have suggested that PB1-F2 may play a role in monocytic cells. To address the potential cell-type specific effects of VN1203 PB1-F2, we infected murine bone marrow derived macrophages (BMDM) and dendritic cells (BMDDC) as a model system for monocytic cells (Fig. 1E). Notably, in macrophages and dendritic cells, we were unable to detect PB1-F2 expression (data not shown), possibly as a result of low protein stability and/or lower levels of protein expression. Interestingly, in both these bone marrow derived cells, the N66S virus appeared to replicate better than the WT and dF2, as estimated by western blot against viral nucleoprotein at 8 and 16 h post-infection.

### VN1203 PB1-F2 Does Not Predominantly Co-Localize with Mitochondria

PB1-F2 was initially described to localize predominantly to mitochondria (as shown for A/Puerto Rico/8/1934 [14]) but also in the cytoplasm and nucleus [39], depending on the virus strain studied. The mitochondrial localization and interaction with mitochondrial membrane proteins VDAC-1 and ANT-3 were proposed to be the basis for the PB1-F2 pro-apoptotic function [18]. Since the N66S substitution is localized in close proximity to the mitochondrial localization sequence, we tested whether this substitution alters the sub-cellular localization of VN1203 PB1-F2 in mouse epithelial cells. By immunofluorescent staining of LA-4 murine lung epithelial cells infected with the WT, dF2 or N66S mutants of VN1203, we could detect WT PB1-F2 as early as 8 h p.i. (Fig. S1A, panel 2). At 4 h p.i. no PB1-F2 specific signal was detectable (data not shown). In agreement with our western blot data the N66S substituted PB1-F2 was not detectable at 8h p.i.. WT PB1-F2 was expressed predominantly in the nucleus, with a few cells showing diffuse staining of the whole cell body. Remarkably, co-staining against the NP of influenza virus revealed that only a minor portion of infected (NP positive) cells showed PB1-F2 staining, underlining the low expression or stability levels of the PB1-F2 peptide. In agreement with our western blot data, the N66S substituted PB1-F2 was not detectable at 8 h p.i. However, at 24 h post infection both WT and N66S variants of PB1-F2 were detectable, although again not all NP positive cells were positive for PB1-F2, especially for the N66S mutant (Fig. S1B). At this time point both variants of PB1-F2 were distributed throughout the cytoplasm and nucleus. To test if the cytoplasmic PB1-F2 is localized at mitochondria, we co-stained the infected LA-4 cells for prohibitin, an inner mitochondrial membrane protein. As shown in Fig. S1C (and enlarged in Fig. S1D), we did not detect predominant co-localization of PB1-F2 WT or N66S with prohibitin, but rather a diffuse distribution throughout the cytoplasm and nucleus.

### N66S Substitution in VN1203 PB1-F2 Reduces Antiviral Responses in Murine Monocytic Cells

**Figure 2:**
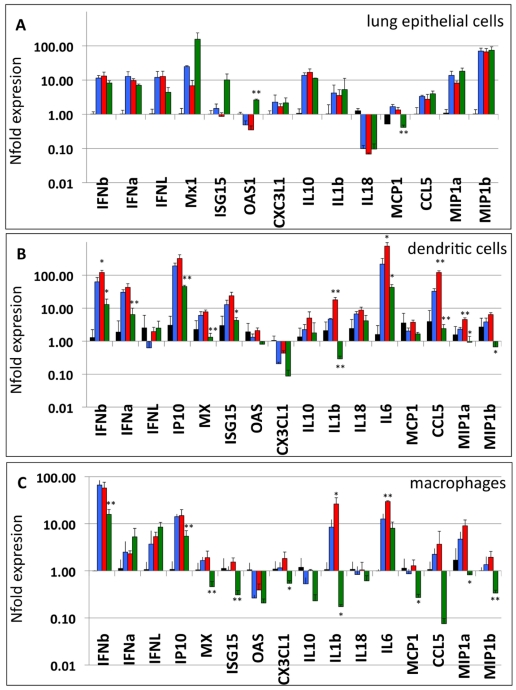
PB1-F2 alters host response against A/Viet Nam/1203/2004 in murine cells. A–C) LA-4 (A), BMDM (B) and BMDDC (C) were infected with 2 MOI of A/Viet Nam/1203/2004 wild type (blue), dF2 (red) or N66S (green). Transcriptional response was measured by gene specific analysis of cDNA amounts from total lysates 8 h post infection. Expression is depicted as mean nfold of mock samples +SD from three independent samples. *P*-values are indicated (*p<0.05, ** *p*<0.01) using WT as standard.

Recent findings implicate an interferon-antagonistic function as a new role for PB1-F2 during viral infection. Using whole genome transcriptome analysis, Connenello et al showed increased levels of type I interferon and interferon stimulated genes (ISGs) in BALB/c mice infected with a recombinant A/WSN/1933 virus containing a A/Hong Kong/483/97 PB1 segment with a N66 PB1-F2 substitution, as compared to a virus containing the S66 PB1-F2 [40]. Thus, we tested whether the substitution (N66S) in a wild type VN1203 H5N1 background would also lead to altered cellular innate responses in LA-4 cells as well as in BMDMs and BMDDCs. In all cell types, we could not detect major differences in the mRNA levels of types I and III interferon induced by VN1203 WT or dF2 virus. Accordingly, the induction levels of ISGs (IP10, MxA, ISG15) did not significantly depend on presence of PB1-F2 (Fig. 2A, B, C). In both BMDMs and BMDDCs, the dF2 virus induced significantly higher levels of proinflammatory cytokines IL1beta and IL6. We could not see these differences in epithelial cells (Fig. 2A). Interestingly, despite higher levels of replication, the N66S mutant virus clearly induced lower levels of interferon dependent antiviral responses in BMDMs and BMDDCs (Fig. 2B and C). Presumably as a consequence of this, mRNA levels of ISGs and cytokines and chemokines were significantly reduced in N66S virus infected BMDMs and BMDDCs. Similar results were seen at early time points of infection in vivo by Connenello and colleagues [40]. Surprisingly, this effect was not visible in LA-4 cells, suggesting a monocytic cell type specific effect of PB1-F2 with regards interferon antagonism.

### PB1-F2 N66S Increases Pathogenesis of Highly Virulent H5N1 in Mice

**Figure 3:**
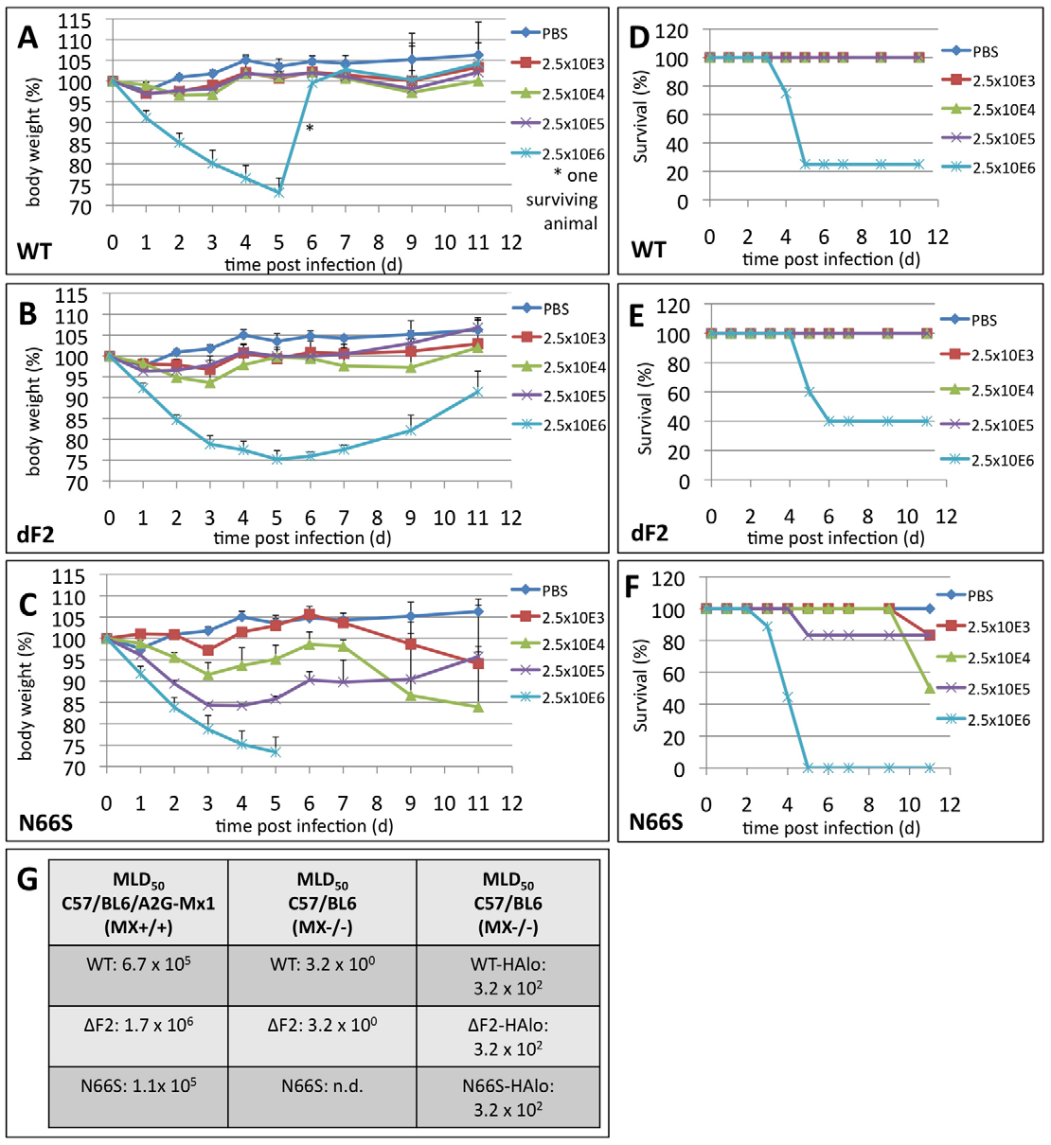
Effects of PB1-F2 expression on viral pathogenesis in vivo. A–C) Weight loss curves of C57/BL/6/A2G-Mx1 mice infected with indicated doses of A/Viet Nam/1203/2004 wild type (A), dF2 (B) or N66S (C). Animals were excluded from analysis when reaching less than 75% initial body weight. Mean %-body weight of 5-9 (initial group size) animals normalized to initial weight +SD is depicted. D–F) Survival curves of C57/BL/6/A2G-Mx1 mice infected with indicated doses of A/Viet Nam/1203/2004 wild type (D), dF2 (E) or N66S (F). %-survival of 5–9 (initial group size) animals is depicted. (G) MLD50 of A/Viet Nam/1203/2004 wild type, dF2 or N66S (high and low pathogenic version) in C57/BL/6/A2G-Mx1 and C57/BL/6 mice.

Next we were interested in determining whether deletion of the PB1-F2 ORF or the N66S substitution would affect viral pathogenesis *in vivo.* The mouse 50% lethal dose (MLD_50_) of recombinant WT VN1203 in C57/BL/6 is ∼3 pfu (John Steel, unpublished data). Since we hypothesized that the N66S substitution might potentially reduce the MLD_50_, (based on the higher replication observed in murine lung epithelial cells), we decided to conduct this experiment in C57/BL/6/A2G-Mx1 mice that express a functional Mx1 gene product. Previous studies have shown a dramatically increased resistance of Mx1^+/+^ mice to both highly pathogenic avian influenza H5N1 viruses and the 1918 pandemic H1N1 virus [38]. As expected, the MLD_50_ for recombinant WT VN1203 was more than 200,000 times higher in C57/BL/6/A2G-Mx1 mice (MLD_50_ = 6.7×10E5 pfu) compared to C57/BL/6 (MLD_50_ = 3.16 pfu) (Fig. 3G). Interestingly, only animals infected with 2.5×10E6 of the wild type virus showed weight loss and decreased survival (Fig. 3A and D). The mice infected with lower doses of virus were completely asymptomatic.

Deletion of the PB1-F2 ORF resulted in a slightly decreased pathogenicity as compared with WT (Fig. 2B, E and G: MLD_50_ = 1.7×10E6 pfu). Interestingly, the N66S substituted virus showed an increased pathogenicity compared to the wild type virus (Fig. 3C and F; MLD_50_ = 1.1×10E5 pfu). Moreover, we observed sustained weight loss, clear signs of sickness (ruffled fur, lethargy) and death in these animals, even in the groups infected with 2.5×10E4 and 2.5×10E3 pfu. Interestingly, in these lower dose infected animals (2.5×10E3 and 2.5×10E4) onset of death occurred unusually late around day 10 post infection. We noted that prior to death, starting at day 9 post infection, 3 out of 6 mice infected with 2.5×10E3 pfu VN1203 N66S showed neurologic symptoms (e.g. hemi-paralysis) indicating a potential viral infection of the central nervous system (CNS). Notably, this was not observed for the WT or dF2 viruses at any dose used. Also, it was not observed in animals infected with higher doses of the N66S virus, likely due to death from pulmonary infection.

Surprisingly, when using a low pathogenic mutant of VN1203 (HAlo) lacking the multi-basic cleavage site, we did not observe differences in lethality (Fig. 3G right panel) or viral lung titers (data not shown) between WT, dF2 or N66S viruses. Given that without the multi-basic cleavage site HAlo viruses are likely to be confined to the respiratory tract, these data suggest that tissue tropism has a role in determining the impact of PB1-F2.

### PB1-F2 N66S Increases H5N1 Viral Replication and Neurotropic Spreading in Mice

**Figure 4:**
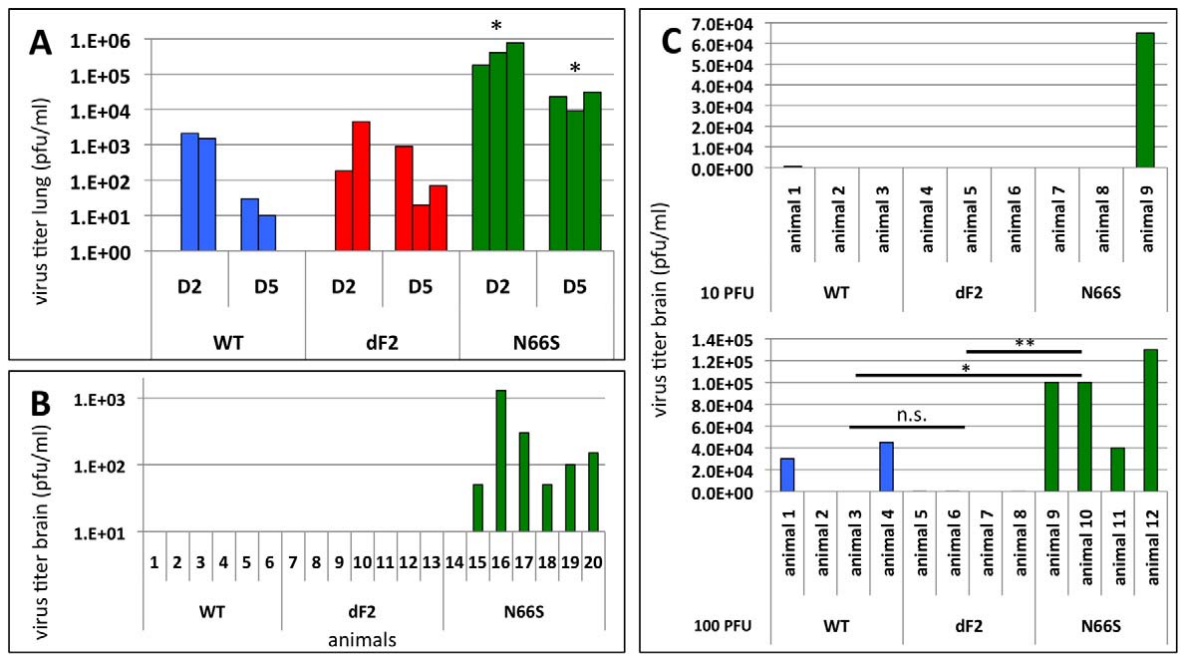
Effects of PB1-F2 expression on viral replication and spreading to the brain in mice. A) C57/BL/6/A2G-Mx1 mice were infected with 2.5×10E6 pfu of VN1203 wild type (blue), dF2 (red) or N66S (green). Viral lung titers were determined on day 2 and 5 post infection in 3 animals by standard plaque assay on MDCK cells. Titers of individual animals (pfu/ml) are depicted, the detect ion limit of the assay was 50 pfu/ml. *P*-values for the mean plaque titers are indicated (*p<0.05) using WT as standard. B) C57/BL/6/A2G-Mx1 mice were infected with 2.5×10E3 pfu of VN1203 wild type (blue), dF2 (red) or N66S (green). Viral brain titers on day 8 post infection of individual animals are depicted. The detection limit of the assay was 50 pfu/ml. C) Direct brain-inoculation of VN1203 wild type (blue), dF2 (red) or N66S (green) (10pfu upper panel, 100pfu lower panel). Viral brain titers (pfu/ml) 48 h post infection are shown for each animal.

In the murine lung epithelial cells (LA-4) the N66S virus replicated to titers 10-fold higher than WT and dF2 (Fig. 1B). Similarly, we observed almost 100-fold higher titers in N66S virus infected mouse lungs on days 2 and 5 post infection, as compared to WT and dF2 infected mice (Fig. 4A). The late neurological symptoms observed in low dose VN1203 N66S virus infected mice suggested spreading of the virus to the CNS at later stages of infection. We thus conducted a separate infection study in C57/BL/6/A2G-Mx1 mice using a sub lethal dose of 2.5×10E3 pfu and measured viral titers in the lung, spleen and brain on day 8 post infection, a day before the onset of neurological symptoms. At this time-point none of the groups showed any detectable levels of virus in the lung (data not shown). As described for highly pathogenic influenza A virus infections in Mx1 positive mice [38], we did not detect systemic spread of WT or dF2 VN1203 into brain or spleen by plaque assay. However, in 6 out of 7 animals infected with N66S virus, we detected virus in the brain (Fig. 4B), but not in spleen or lung (data not shown), suggesting a specific function of N66S PB1-F2 in neurotropism. To further show the replication advantage of the N66S substituted virus in neuronal tissue, we infected mice intracranially with either 10 or 100 pfu and measured viral titers in the brain 2 days post infection (Fig. 4C). The N66S substituted virus replicated significantly better in brain tissue compared to the WT or dF2 viruses in the mice inoculated with 100 pfu (Fig. 4C lower panel). We even observed replication of N66S virus in one out of three animals inoculated with 10 pfu. It should be mentioned that 2 out of 4 animals of the WT infected group showed viral brain titers, but this difference was not significant compared to the PB1-F2 deficient virus infected mice.

In summary, substitution of serine for asparagine at position 66 of PB1-F2 enhances replication of VN1203 in murine in vitro models and reduces the IFN response in infected murine monocytes. In an in vivo mouse model, this substitution enhances pathogenicity, replication and neurotropism. However, we could not detect major differences in viral lung titers and only a mildly altered LD_50_ in mice when comparing the WT and PB1-F2 deficient viruses. Nevertheless, we observed enhanced mRNA levels of the proinflammatory cytokines IL-6 and IL-1b in dF2 virus infected monocytic cells.

### Expression of PB1-F2 Does Not Alter Viral Replication in Duck Cells in vitro

**Figure 5:**
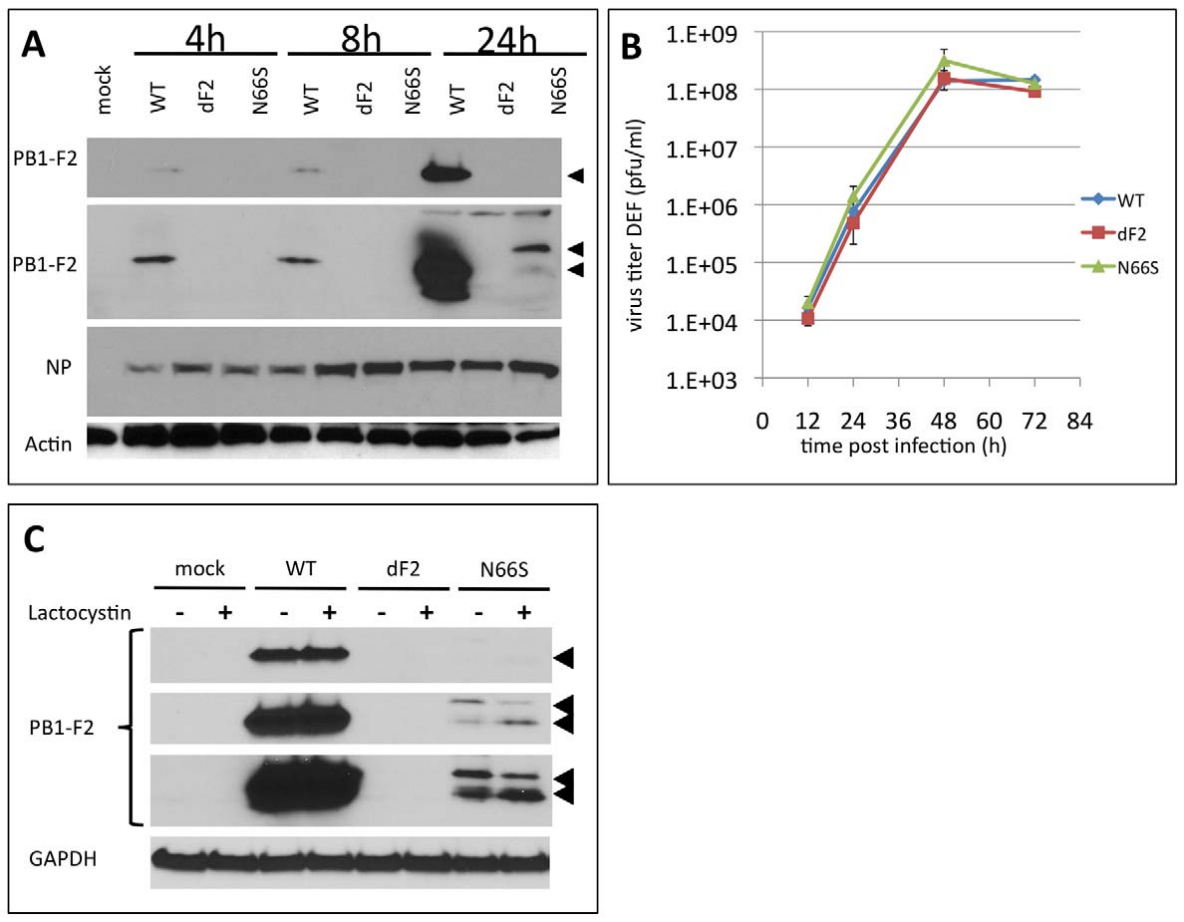
Effects of PB1-F2 expression on replication of A/Viet Nam/1203/2004 in duck fibroblasts *in vitro*. A) Time course of viral protein expression. Duck embryonic fibroblasts (DEF) were infected with 2MOI of A/Viet Nam/1203/2004 wild type, dF2 or N66S. Cells were lysed after indicated time points. Whole cell extracts were analyzed by western blot with specific antibodies against PB1-F2 (upper two panels, short and long exposure, respectively), viral nucleoprotein (NP) or beta-Actin. B) PB1-F2 expression in presence or absence of 10 µM lactocystin. DEF were infected with 2MOI of A/Viet Nam/1203/2004 wild type, dF2 or N66S. Cells were lysed after 24 h. Whole cell extracts were analyzed by western blot with specific antibodies against PB1-F2 (upper three panels, short, medium and long exposure, respectively, PB1-F2 is indicated by black arrows), equal loading is indicated by GAPDH levels (lower panel). C) Multi-cycle growth curve of A/Viet Nam/1203/2004 wild type (blue), dF2 (red) or N66S (green) on DEF. Cells were infected with 0.05MOI of virus. Viral supernatants were taken at the indicated time points. Viral titers were determined by standard plaque assay on MDCK in absence of trypsin. Mean pfu/ml of independent triplicates ±SD indicated by the error bar are depicted.

The PB1-F2 ORF is highly conserved in viruses isolated from ducks. More than 95% of viruses express a PB1-F2 of 87 amino acids or longer (NCBI Influenza virus resource: http://www.ncbi.nlm.nih.gov/genomes/FLU/FLU.html and Influenza Research Database (IRD): http://www.fludb.org/brc/home.do?decorator=influenza). We thus hypothesized a specific need to retain PB1-F2 in an avian host environment and consequently envisaged a different outcome to our infection study in an avian host as compared to a mammalian host. To test the growth properties of the three recombinant VN1203 viruses in avian cells we first performed infection experiments in duck fibroblast cells (DEF). As shown for the murine lung epithelial cells, the N66S mutant protein was present in lower amounts in infected cells and expression levels increased in the presence of the proteasome inhibitor lactocystin (Fig. 5B).

In stark contrast to the murine cell models, we did not observe any increase in viral replication or viral NP levels by substituting serine for asparagine at position 66 of VN1203 PB1-F2 (Fig. 5A and B). Similar to what we observed in the murine lung cell line, in the infected DEFs the WT PB1-F2 is expressed to higher levels (already detectable after 4 h post infection, Fig. 5A), than the N66S variant (first detectable after 24 h). Interestingly, the N66S PB1-F2 shows a slower migrating form in western blot, as shown in murine cells, which may indicate a post-translational modification. Of note, the proteasome inhibition mainly affected the levels of low molecular weight PB1-F2, again indicating that the higher molecular weight form could be protected from degradation (Fig. 5B).

As shown in LA-4 the polyclonal serum raised against the N-terminus of PB1-F2 detects a higher migrating band only present in infected duck cells (24 h post infection).

Next we examined the localization of PB1-F2s in infected DEFs. The WT PB1-F2 was localized mainly to the nucleus at 8 h pi (Fig. S2A). Similar to infected LA-4 cells, not all NP positive cells were also positive for PB1-F2. At 24 h post infection, we could detect both the WT and the N66S PB1-F2 distributed throughout the whole cell (Fig. S2B). Co-staining for the mitochondrial protein prohibitin did not reveal predominant mitochondrial localization of WT or N66S PB1-F2 (Fig. S2C and enlarged in Fig. S2D) as observed previously in LA-4 cells. This suggests that the inability of this PB1-F2 to localize to mitochondria is not a species-specific phenomenon.

### Deletion of PB1-F2 Reduces Pathogenicity of H5N1 in Ducks

**Figure 6:**
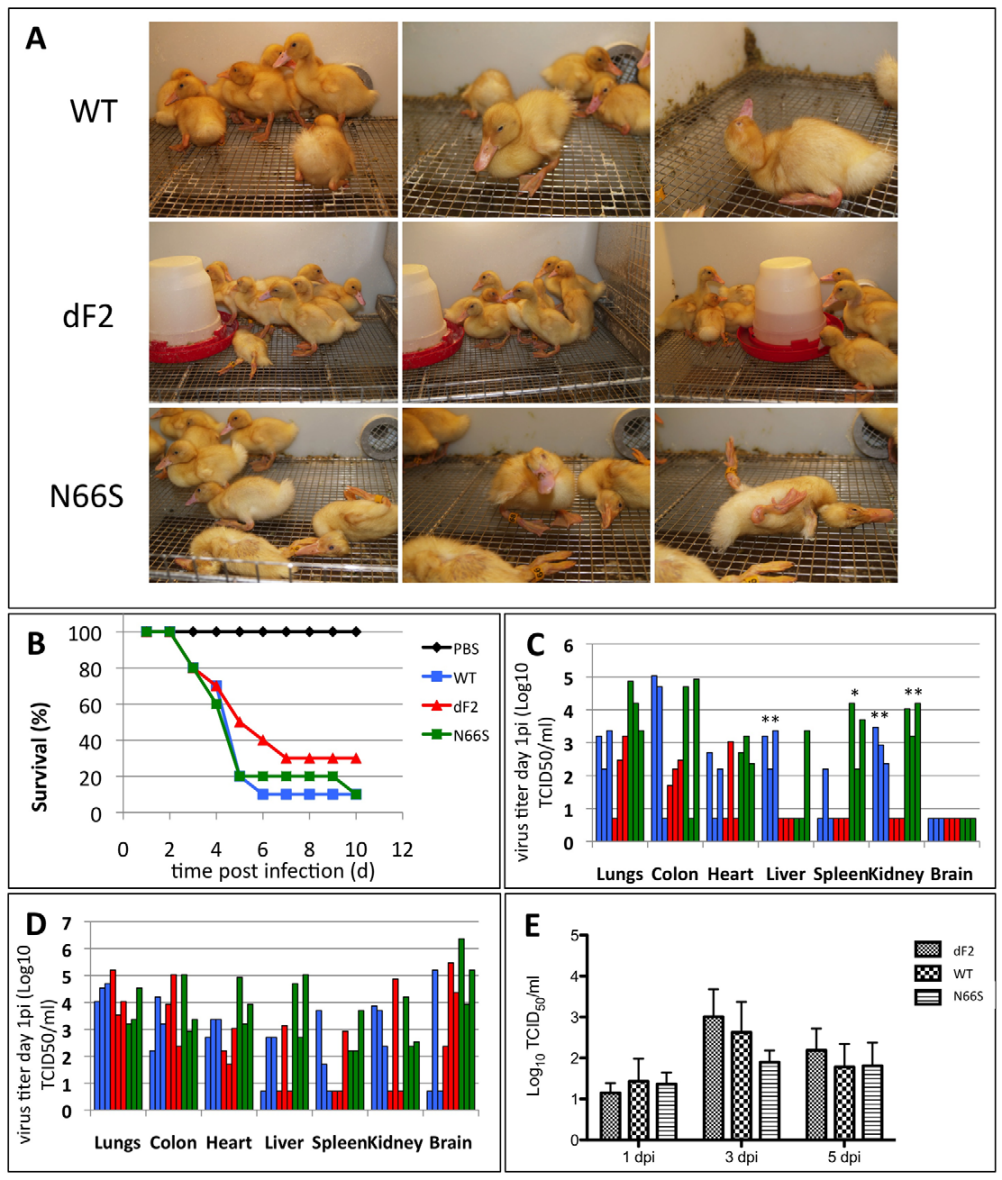
Diminished pathogenicity of PB1-F2 deficient virus in ducks. Ten 2-week old white Peking ducks were infected with 10E4 pfu of A/Viet Nam/1203/2004 wild type, dF2 or N66S. A) Three representative pictures of infected ducks were taken on day 4 p.i.. B) Survival curves of 10 2-week old white peking ducks were infected with 10E4 pfu of A/Viet Nam/1203/2004 wild type (blue), dF2 (red) or N66S (green). Survival (%) is depicted. C and D) Viral titers of indicated organs from day 1 (C) and day 3 (D) post infection with wild type (blue), dF2 (red) or N66S (green). TCID_50_/ml of organs from individual animals are depicted. E) Mean viral titers (TCID_50_/ml) cloacal swabs from 10 animals per group (initial group size) are depicted. *P*-values are indicated (*p<0.05, ** *p*<0.01) using dF2 as standard.

To address a potential impact of PB1-F2 deletion or N66S substitution in vivo, we next conducted pathogenicity studies in two-week-old white Peking ducks. The infected ducks were monitored for clinical signs and scored daily as described previously [32], [33]. In the first two days of infection, we could clearly detect differences in the onset of clinical signs between the three groups of animals (Table 2 and Fig. 6A and B). The WT virus infected animals show sickness (n = 7) or severe sickness (n = 3) by day 2 of infection (pathogenicity index (PI) of 2.28). In contrast the N66S virus infected ducks show a slightly faster disease progression (day 1: sick n = 9, severely sick n = 1, (PI = 2.4)). However, both viruses efficiently killed the ducks (9/10 animals) after 10 days of infection. In contrast, the dF2 virus infected animals progressed showed significantly slower progression of clinical signs of sickness (day 2: sick n = 1, (PI = 1.66) as compared to the WT and N66S virus infection. Furthermore, the dF2 virus infected animals showed a greater survival rate (3/10), although this difference is not statistically significant. It has to be pointed out that all three viruses clearly have a pathogenicity index of >1.2, defining each as a highly pathogenic virus in birds.

**Table 2:** Pathogenicity-Index of VN1203 WT, dF2 and N66S in white peking ducks.

Group	1*	2	3	4	5	6	7	8	9	10	Total	Weight	Sum	Index**
**PBS**														
Healthy	10	10	10	10	10	10	10	10	10	10	100	0	0	**0**
Sick	0	0	0	0	0	0	0	0	0	0	0	1	0	
Severely sick	0	0	0	0	0	0	0	0	0	0	0	2	0	
Dead	0	0	0	0	0	0	0	0	0	0	0	3	0	
Total											100		0	
**WT**														
Healthy	7	0	0	0	0	0	0	0	0	1	8	0	0	**2.28**
Sick	3	7	0	2	0	0	0	1	1	0	14	1	14	
Severely sick	0	3	8	5	2	1	1	0	0	0	20	2	40	
Dead	0	0	2	3	8	9	9	9	9	9	58	3	174	
Total											100		228	
**dF2**														
Healthy	9	9	0	0	0	0	0	0	3	3	24	0	0	**1.66**
Sick	1	1	8	6	5	3	3	3	0	0	30	1	30	
Severely sick	0	0	0	1	0	1	0	0	0	0	2	2	4	
Dead	0	0	2	3	5	6	7	7	7	7	44	3	132	
Total											100		166	
**N66S**														
Healthy	0	0	0	0	0	0	0	0	0	0	0	0	0	**2.40**
Sick	9	6	0	0	0	0	0	0	0	0	15	1	15	
Severely sick	1	4	8	6	2	2	2	2	2	1	30	2	60	
Dead	0	0	2	4	8	8	8	8	8	9	55	3	165	
Total											100		240	

*days post infection.

**calculated as previously described [32].

### Expression of WT and N66S PB1-F2 Accelerates Systemic Spreading of VN1203 in Ducks but Does Not Influence Shedding

To test if PB1-F2 plays a role in viral dissemination to organs in infected ducks, we measured viral titers in different organs at various time post infection. By day 1 post infection TCID_50_ in colon, liver, spleen and kidney are higher in WT and N66S virus infected animals compared to dF2 virus infected animals (Fig. 6C). However, likely due to the use of outbred animals, the variation within each group was high and by day 3 post infection no statistically significant differences were detectable (Fig. 6D). Surprisingly, we did not observe enhanced viral titers in cloacal swabs of WT or N66S virus infected ducks compared to dF2 virus infected animals (Fig. 6E and F) implicating similar shedding rates. In ducks, we could not detect different levels of type I interferon mRNA on day 1 or 3 post infection in spleen, lung or colon of the three groups of infected animals (data not shown). Thus it is possible, at least for VN1203, that PB1-F2 has an interferon-antagonist independent role in enhancing pathogenicity in avian hosts.

Overall, we observed that the VN1203 WT and N66S viruses behave very similarly in ducks with respect to replication and pathogenicity. Both viruses spread systemically to spleen and kidney by day 1 post infection and onset of severe clinical signs occurs by day 1 or 2 post infection. In contrast the deletion of PB1-F2 resulted in delayed onset of clinical symptoms and systemic spread of the virus is first detectable only by day 3 post infection. Our data suggest that the full length PB1-F2 ORF is important for rapid systemic viral dissemination of HPAIV in birds. This contrasts with our mammalian host data, in which deletion of PB1-F2 has only a minor impact on replication and pathogenicity, although variations at position 66 can enhance virulence.

## Discussion

HPAIV infections are associated with hyper-production of inflammatory cytokines and chemokines, systemic viral spreading and severe multi-organ damage in both mammalian and avian hosts. The viral and host factors contributing to this unusually severe outcome have been studied for years, but are still not completely identified or understood. Here we analyzed the impact of PB1-F2, a non-structural viral protein, on viral pathogenesis and host response in mammalian and avian model systems. PB1-F2 is present in the majority of avian isolates of all subtypes, but the full length open reading frame is lost over time in many mammalian isolates. To our knowledge this is the first comparative study using an iso-genic approach to test the function of PB1-F2 in a HPAIV in birds and mammals. We further analyzed the impact of a N66S substitution in PB1-F2, which was previously shown to be associated with viral pathogenesis of the 1918:H1N1 pandemic virus [22] and that was recently proposed to promote interferon antagonistic properties of PB1-F2 in mice [40].

In the murine cell system, the three tested viruses showed differences in growth and host response depending on the cell model used. In monocytic cells (BMDMs and BMDDCs) the N66S substituted virus replicated faster as indicated by higher levels of NP and induced lower levels of type I interferon, ISGs and proinflammatory cytokines and chemokines. Interestingly, the PB1-F2 deleted virus induced higher levels of IL-6 and IL-1beta in monocytic cells. In contrast, in lung epithelial cells the host response was similar among the three viruses. A cell type specific function of PB1-F2 was already proposed when PB1-F2 was initially described ∼10 years ago. Chen et al provided experimental evidence for a pro-apoptotic function of PR8 PB1-F2 that predominantly affects monocytes [14]. We found that the tested VN1203 PB1-F2 used in this study does not predominantly localize to mitochondria. Chen et al showed, that two leucine residues in the C-terminus of PB1-F2 are essential for mitochondrial localization of PB1-F2 (L69 and L75) [39]. They could show that PB1-F2 of A/Hong Kong/156/1997 does not localize to mitochondria due to alternative amino acids at position 69 and 75 (Q69 and H75). Interestingly substitution of these residues with leucine changes the subcellular localization of PB1-F2 to the mitochondria, but does not affect virus induced apoptosis levels in vitro or viral pathogenicity in mice when tested in a 7+1 PR8 system with segment 2 of A/Hong Kong/156/1997. Notably, PB1-F2 of VN1203 does not contain the essential leucines for mitochondrial localization at position 69 (69Q) and 75 (75R), which is in full agreement with what we have observed by immunofluorescence in infected murine and duck cells. It is thus unclear if VN1203 shares the published pro-apoptotic function of PB1-F2s from other viruses (e.g. PR8) or if the cytoplasmic PB1-F2 can still interact with mitochondrial host factors such as VDAC and ANT3 [18].

When PB1-F2 was initially described [14], it was shown that this short polypeptide is highly unstable and degraded in proteasome dependent way. We did not observe changes in the levels of WT PB1-F2 when blocking proteasome function. However, we show here in murine and duck cells that the protein levels of the N66S mutant can be increased by exposing the infected cells to lactocystin. This increase mainly affects the faster migrating protein species and could implicate that a modified version of PB1-F2 N66S is protected from proteasomal degradation. At this point it is unclear if and how the stability of N66S affects its function in increasing pathogenicity of the virus.

We consistently observed an increase of viral proteins and/or viral titers in the N66S virus infected murine cells. Mazur et al have shown that PB1-F2 of PR8 can interact with the polymerase subunit PB1 and enhance polymerase function. Nevertheless, this does not affect viral replication and pathogenicity in vitro and in vivo [20], [21]. McAuley et al could show that PB1-F2 of VN1203 can increase the polymerase function of a PR8 derived polymerase complex in human 293T [21]. However, it did not affect pathogenesis of a recombinant PR8 with a VN1203 PB1-F2 in mice. Nevertheless, none of these studies addressed a potential effect of sequence variations at position 66. In our mouse fibroblast system (3T3 cells), we did not detect changes in viral polymerase function in the presence or absence of PB1-F2. Moreover the N66S substitution did not enhance viral polymerase activity in this in vitro assay, as could be suspected from the enhanced replication and enhanced viral protein levels. This is contradictory to the data presented by McAuley et al. However, it has to be pointed out that this group combined PR8 polymerase subunits with PB1-F2 from different viruses [21]. We cannot exclude that the PR8 polymerase function is specifically modulated by certain PB1-F2s.

We clearly observed enhanced replication of the N66S substituted virus in vitro and in vivo, resulting in increased pathogenicity and higher lung titers in mice, consistent with earlier work using a 7+1 influenza A/WSN/33 virus with an HK/483 PB1 segment [22]. Interestingly, the N66S variant was the only virus that was able to spread to the CNS of Mx1 positive mice, leading to detectable viral brain titers 8 days post infection. This infection of the CNS is most likely responsible for the observed paralysis in mice infected with low doses of virus and the unusual second wave of deaths in infected mice around day 10 of infection. When Mx1 positive mice were initially infected with H5N1 and the 1918 H1N1 virus [38], no systemic replication could be detected. We also did not detect systemic viral spreading after infection with low doses of WT H5N1 virus. Mechanistically it is not clear at this point how PB1-F2 potentiates spread of the N66S virus to the central nervous system. It is also intriguing that we only detected virus in the brain and not in other organs. Direct viral inoculation of the brain resulted in sustained replication of the N66S substituted virus, while the WT and dF2 mutant did not replicate or replicated poorly. This implies that PB1-F2 N66S supports neuronal dissemination of the virus, and whether this is due to its specific interaction with a host factor in the brain remains to determined. Very recently Shinya et al investigated neuropathogenicity of different H5N1 influenza A virus isolates in ferrets [41]. Interestingly the authors could show that influenza A/Hong Kong/483/1997, which contains a serine at position 66 of PB1-F2 disseminates wider throughout the brain tissue, most likely by infecting vascular cells, since severe vascular lesions were observed. These lesions were not found in ferrets infected with a closely related isolate, A/Hong Kong/486/1997, which contains an asparagine at positione 66 of PB1-F2. It remains elusive if the observed phenotype is based on the N66S substitution or other changes between the two isolates.

To our surprise, we did not observe enhanced viral replication or pathogenicity of the N66S virus when using a low pathogenic mutant background of VN1203 lacking the multi-basic cleavage site in HA. The multi-basic cleavage site is essential for systemic spreading of HPAIV in mammals, allowing HPAIV to replicate independently of lung resident extracellular proteases [32]. It appears then that both a multi-basic cleavage site in HA and a S66 amino-acid residue in PB1-F2 are required to achieve high levels of replication and spread to the brain in Mx1 competent mice. At this juncture, we cannot exclude that the enhanced lethality of the N66S mutant virus and the neurotropism in mice are linked. Newly developed viral tracking systems, may allow us to find more details on the type of cells infected by these viruses *in vivo* [42].

In the Mx1 positive mouse system, the expression of a PB1-F2 with a serine at position 66 clearly enhances viral replication and pathogenicity *in vivo.* Interestingly, deletion of the PB1-F2 ORF had little effect on replication or lethality of the virus, when compared to the WT virus. This was described earlier for PR8 virus, where the deletion of PB1-F2 did not affect replication and lethality in mice [24]. In the human H1N1 isolates, the full length PB1-F2 ORF is lost by introduction of a premature stop codon at position 59, [25]. This could indicate, that full length PB1-F2 per se is not essential for viral fitness in humans/mammals and could explain why the deletion of PB1-F2 had no major effect on pathogenicity of the viruses tested here and in other studies [21], [24]. Accordingly, the currently circulating swine origin H1N1 virus has no functional PB1-F2 ORF, and is nevertheless spreading successfully [26]. At this point, the function of a C-terminally truncated PB1-F2 in humans remains elusive. PB1-F2 is expressed from a +1 ORF of segment 2 of IAV. Besides PB1 and PB1-F2 this segment also encodes for N40, an N-terminally truncated version of PB1 that was first described in 2009 [43]. Deletion of the PB1-F2 ORF, by mutation of the start AUG enhances expression of N40 as a consequence of ribosomal shifting. Deletion of N40 results in attenuation of the virus, but this also changes the PB1 sequence. In contrast, enhanced expression of N40, as shown for PB1-F2 deficient virus mutants, has no effect on viral replication in vitro and in ovo [43]. Thus, the function of N40 remains elusive. We cannot exclude the possibility that in our systems deletion of the PB1-F2 ORF might cause phenotypes that are indirectly influenced by the levels of N40.

Despite the lack of differences in replication in vitro and in vivo or major impact on pathogenicity in mice, we observed increased mRNA levels of IL1beta and IL6 in murine BMDMs and BMDDCs infected with PB1-F2 deficient viruses in vitro. So far, no correlations have been made between PB1-F2 and inflammatory responses. It is striking that this effect is more pronounced in monocytic cells, since these cells have previously been shown to be more susceptible to the pro-apoptotic function of PB1-F2 [14]. In contrast to the wildtype and PB1-F2 deficient virus, the N66S virus induces significantly lower amounts of type I and type III interferon and consequently ISGs in BMDM and BMDDCs. Recent work showed that in a WSN/33 virus encoding the HK/483 PB1, the N66S substitution in the PB1-F2 ORF resulted in reduced type I interferon levels by day one of infection, despite higher viral titers [40]. Since pDCs and alveolar macrophages are the main producers of type I interferon and proinflammatory cytokines, it is possible that PB1-F2 primarily acts in these cells by a yet undetermined mechanism. In parallel to this work, a study by Varga et al (accepted for publication), showed that PB1-F2 expression can decrease RIG-I and MAVS induced IFN-β production. The detailed mechanism is still unknown, but PB1-F2 seems to act at the level of MAVS. It is so far unclear to what extent PB1-F2 contributes to antagonism of the type I interferon response and why deletion of PB1-F2 (as in the pandemic 2009 H1N1 strains) does not result in diminished viral replication.

In ducks, deletion of PB1-F2 had a more striking effect on pathogenesis. We clearly observed a delayed onset of clinical symptoms in the animals infected with the PB1-F2 deficient virus. Moreover, the systemic spreading of the virus was significantly delayed, when compared to the wild type and N66S virus. Nevertheless, the deletion of PB1-F2 did not reduce lethality to the levels of low pathogenic avian viruses. Moreover, we did not observe statistically significant differences in the type I interferon levels present in infected animals. Although this could be a consequence of the high inner group variation, due to the outbread nature of the animals, it may also be that the proposed anti-interferon function of PB1-F2 is specific for mammals. We are currently testing this hypothesis.

We also did not see major differences in viral loads in the cloacal swabs of these animals, indicating that PB1-F2 does not affect viral shedding. A recent study by Marjuki et al has shown that three point mutations in PB1-F2 of VN1203 can affect polymerase function in chicken cells and pathogenicity in mallard ducks [37]. Interestingly, these three mutations change the PB1-F2 of the highly pathogenic VN1203 into the PB1-F2 of the low pathogenic A/chicken/Vietnam/C58/04. The same group showed in a previous study that the PB1 segment essentially contributes to pathogenicity of VN1203 in birds [44]. Taken together, these data and our data imply that PB1-F2 is important for the pathogenicity of H5N1 HPAIV. Moreover, the high conservation of PB1-F2 in avian isolates suggests a yet to be determined, but essential, function of PB1-F2 for these viruses.

It is intriguing that the N66S substitution of PB1-F2, a polymorphism associated with increased pathogenicity of the 1918 H1N1 and the highly pathogenic H5N1 isolates from Hong Kong 1997, significantly enhances the pathogenicity of VN1203 in mice, but has little detectable effect in ducks. Intriguingly, in many avian isolates the serine at position 66 is common. This could mean that the molecular mechanism for the enhanced pathogenicity of viruses with this substitution in mammals does not apply to avian hosts.

In summary, our study shows that PB1-F2 is an important, evolutionary conserved pathogenicity factor of HPAIV in avian species that supports faster viral spreading into different organs. In mammals the 1918:H1N1-like N66S substitution supports spreading and replication in the CNS, by a yet to be defined mechanism. The increase in viral pathogenicity of N66S mutant is possibly due to inhibition of type I interferon and proinflammatory responses in monocytic cells.

## Supporting Information

Figure S1 **Subcellular localization of PB1-F2 in LA-4 during viral infection.** LA-4 were infected for 8h (A) or 24h (B) with 2 MOI of A/Viet Nam/1203/2004 wild type, dF2 or N66S. Nuclei were stained with DAPI (blue), PB1-F2 was stained with a polyclonal rabbit serum and anti rabbit-Alexa-488 (green) and NP was stained with a monoclonal mouse antibody and anti-mouse-Alexa-555 (red). Merged pictures are shown in the right panel. All pictures were taken with a 40x objective using identical exposure settings for the green and red channel. Representative cells are shown. C) LA-4 were infected for 24h as described for A/B. Nuclei were stained with DAPI (blue), PB1-F2 was stained with a polyclonal rabbit serum and anti rabbit-Alexa-488 (green) and prohibitin was stained with a monoclonal mouse antibody and anti-mouse-Alexa-555 (red). All pictures were taken with a 63x objective using identical exposure settings for the green and red chanel. D) enlarged version of VN1203 WT and N66S from Fig. S1C.
(TIFF)

Figure S2 **Subcellular localization of PB1-F2 in duck embryonic fibroblasts during viral infection.** DEF were infected for 8h (A) or 24h (B) with 2 MOI of A/Viet Nam/1203/2004 wild type, dF2 or N66S. Nuclei were stained with DAPI (blue), PB1-F2 was stained with a polyclonal rabbit serum and anti rabbit-Alexa-488 (green) and NP was stained with a monoclonal mouse antibody and anti-mouse-Alexa-555 (red). Merged pictures are shown in the right panel. All pictures were taken with a 40x objective using identical exposure settings for the green and red channel. Representative cells are shown. C) DEF were infected for 24h as described for A/B. Nuclei were stained with DAPI (blue), PB1-F2 was stained with a polyclonal rabbit serum and anti rabbit-Alexa-488 (green) and prohibitin was stained with a monoclonal mouse antibody and anti-mouse-Alexa-555 (red). All pictures were taken with a 63x objective using identical exposure settings for the green and red chanel. D) enlarged version of VN1203 WT and N66S from Fig. S2C.
(TIFF)
