# Supplementary figures and images for: Differential Contribution of PB1-F2 to the Virulence of Highly Pathogenic H5N1 Influenza A Virus in Mammalian and Avian Species
Source: PLoS Pathog. 2011 Aug 11;7(8):e1002186. doi: 10.1371/journal.ppat.1002186 (PMC3154844; doi:10.1371/journal.ppat.1002186)

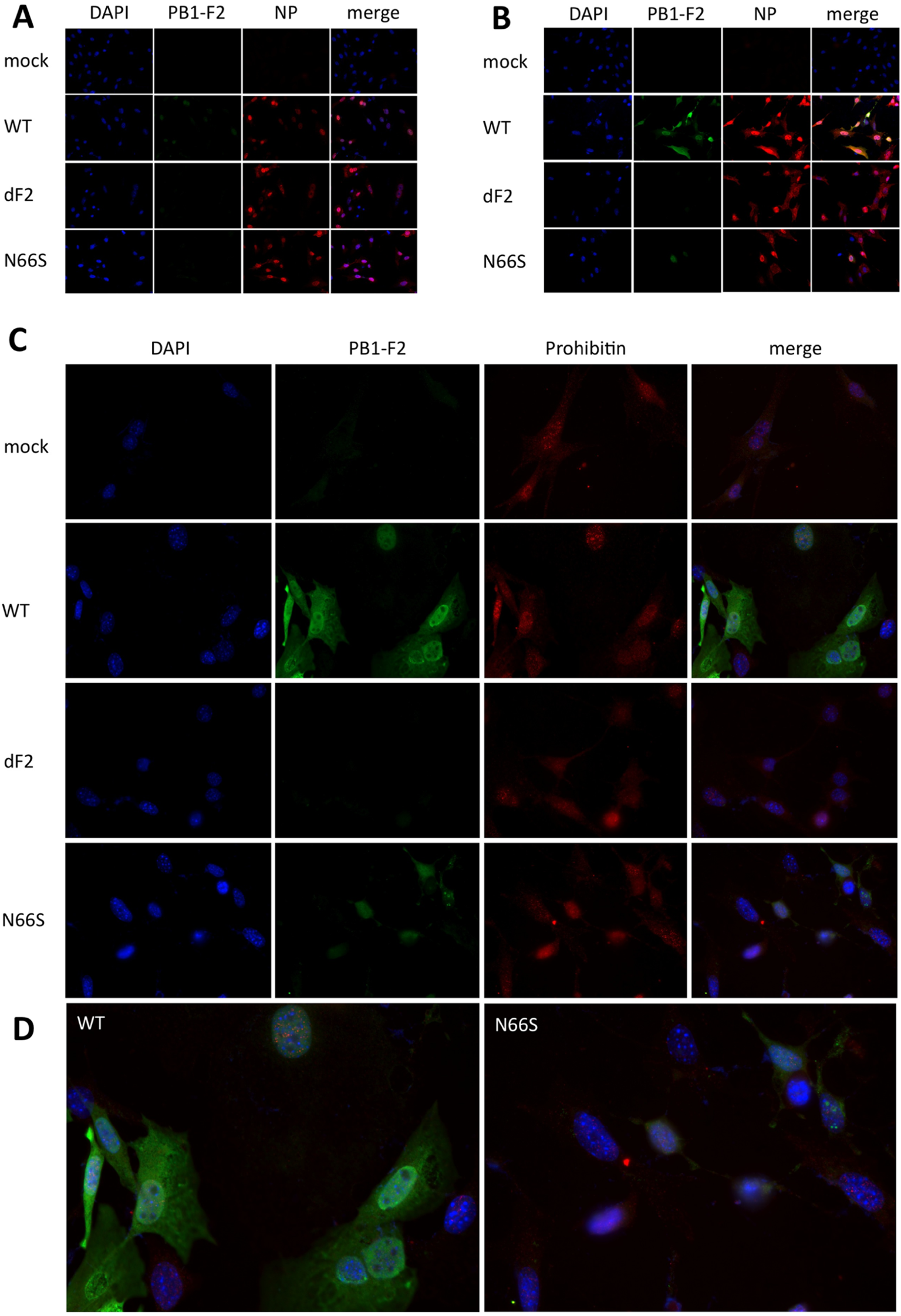

Supplement: Figure S1 — Subcellular localization of PB1-F2 in LA-4 during viral infection. LA-4 were infected for 8h (A) or 24h (B) with 2 MOI of A/Viet Nam/1203/2004 wild type, dF2 or N66S. Nuclei were stained with DAPI (blue), PB1-F2 was stained with a polyclonal rabbit serum and anti rabbit-Alexa-488 (green) and NP was stained with a monoclonal mouse antibody and anti-mouse-Alexa-555 (red). Merged pictures are shown in the right panel. All pictures were taken with a 40x objective using identical exposure settings for the green and red channel. Representative cells are shown. C) LA-4 were infected for 24h as described for A/B. Nuclei were stained with DAPI (blue), PB1-F2 was stained with a polyclonal rabbit serum and anti rabbit-Alexa-488 (green) and prohibitin was stained with a monoclonal mouse antibody and anti-mouse-Alexa-555 (red). All pictures were taken with a 63x objective using identical exposure settings for the green and red chanel. D) enlarged version of VN1203 WT and N66S from Fig. S1C. (TIFF) [file ppat.1002186.s001.tiff]

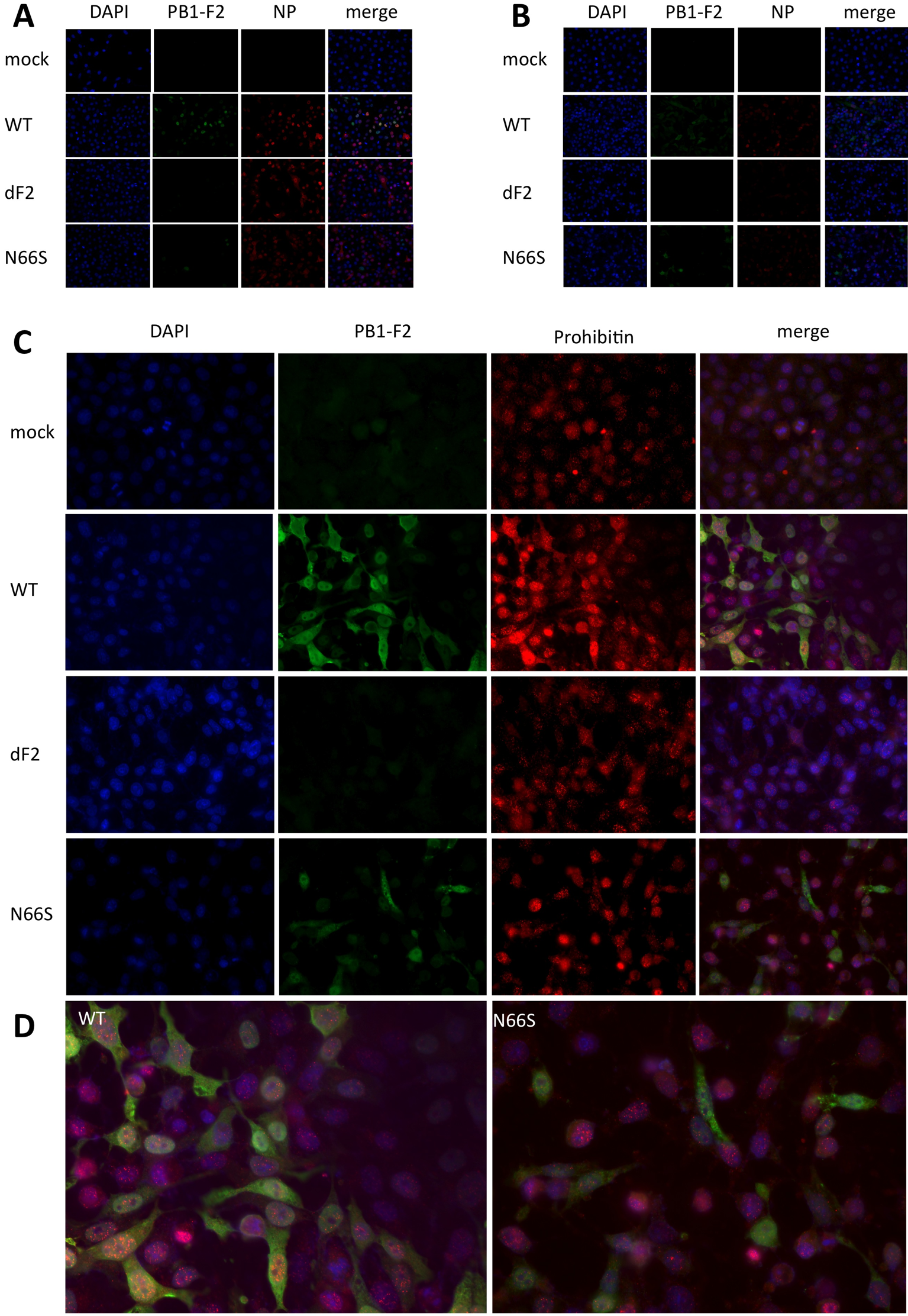

Supplement: Figure S2 — Subcellular localization of PB1-F2 in duck embryonic fibroblasts during viral infection. DEF were infected for 8h (A) or 24h (B) with 2 MOI of A/Viet Nam/1203/2004 wild type, dF2 or N66S. Nuclei were stained with DAPI (blue), PB1-F2 was stained with a polyclonal rabbit serum and anti rabbit-Alexa-488 (green) and NP was stained with a monoclonal mouse antibody and anti-mouse-Alexa-555 (red). Merged pictures are shown in the right panel. All pictures were taken with a 40x objective using identical exposure settings for the green and red channel. Representative cells are shown. C) DEF were infected for 24h as described for A/B. Nuclei were stained with DAPI (blue), PB1-F2 was stained with a polyclonal rabbit serum and anti rabbit-Alexa-488 (green) and prohibitin was stained with a monoclonal mouse antibody and anti-mouse-Alexa-555 (red). All pictures were taken with a 63x objective using identical exposure settings for the green and red chanel. D) enlarged version of VN1203 WT and N66S from Fig. S2C. (TIFF) [file ppat.1002186.s002.tiff]
